# In Memoriam, Byron H. Daggett, M. D.

**Published:** 1904-02

**Authors:** Nelson W. Wilson


### In Memoriam, Byron H. Daggett, M. D.

BY NELSON W. WILSON, M. D.

The arrogance and the fatefulness of death is seldom made so apparent as in the battle which ended in the passing of Byron H. Daggett from among those who are, to that vast army which has fallen to the embrace of the Great Destroyer.

We, whose daily life brings us into frequent conflict with death, are wont to be looked upon by an unthinking lay mind as careless; that our familiarity with the supreme moment of transformation when the light of the world pales into darkness before the luster of the hereafter, brings us to gaze with impassivity of death. Yet, there is not one who knew him even casually, who did not experience a sense of genuine sorrow when announcement was made of the death of Dr. Daggett. Those of his profession who were so fortunate as to enjoy his intimate friendship in recent years, knew that he was battling even then with a pathologic condition which, though it gave him pain,—agonising, excruciating pain,—that would have warped and twisted a less rugged character into a life of perpetual irritability, was manfully endured, and that he was silently fighting against it with all the valor of a strong and uncomplaining soul. Who knows of the sufferings of this man, the secret sufferings as the days went by? Publicly he spoke of it rarely and then lightly; yet in the privacy of his life he suffered; and, suffering, he hid his agony beneath a smile and went his way among men doing good. If there are those who, misunderstanding him, or viewing him on the surface, bethought him of sharp tongue or biting epigram, they were unfortunate in their lack of cultivation of a broad-minded gentleman, of strong personality, sturdy friendship and fearless character; they saw the fleeting clouds, mayhap, of a pain-storm; they foreswore to gaze upon the beauties of the rainbow.

Beneath the rugged exterior of this man whom Death has conquered there was a smile and perpetual sunshine for those who sought his friendship; a willing hand for those who sought his aid, and a broad experience and a ripe judgment for those who sought his advice. His charities and his virtues were manifold and lasting; his faults were as the dew of the morning, forgot in the sunshine of a perfect day. The memory of man sees only the faultless luster, the beauties of the colorfulness of the gem of life; its tiny flaws, if flaws there be, are not for the mind's eye of man. The gem is set in the diadem of Death—a spoil of war.

Dr. Daggett's life was a busy one. No laggard was he. Ever in the forefront of the progress of medicine he did much to upbuild the profession. He kept pace with the world's research, and in spite of his many hospital services, and the confining hours of his private practice, he found time to enrich the literature of the surgery of the genitourinary system by his contributions of original research. Whatever his coworkers in the specialty which he practised may have thought of his methods, they admired his originality and gave him credit for his painstaking labors, and his name appears frequently in the textbooks on genitourinary diseases as the originator of methods of operative procedures and treatments. He devised instruments for urethral work which have become valuable aids in diagnosis and treatment, and the operating table, which bears his name, is ample evidence of his inventive genius. He was a bold surgeon, a gentle physician, a kindly man. Such is his epitaph. It is graven on the tablets of our memory, for he was our friend.

At the conclusion of the memorials, Dr. Wende introduced Dr. Krauss as the new president of the society. Dr. Krauss briefly thanked the members, and adjournment followed.

